# AMPK activation attenuates inflammatory pain through inhibiting NF-κB activation and IL-1β expression

**DOI:** 10.1186/s12974-019-1411-x

**Published:** 2019-02-12

**Authors:** Hong-Chun Xiang, Li-Xue Lin, Xue-Fei Hu, He Zhu, Hong-Ping Li, Ru-Yue Zhang, Liang Hu, Wen-Tao Liu, Yi-Lin Zhao, Yang Shu, Hui-Lin Pan, Man Li

**Affiliations:** 1 Department of Neurobiology, School of Basic Medicine, Tongji Medical College, Huazhong University of Science and Technology, 13 Hangkong Road, Wuhan, 430030 Hubei People’s Republic of China; 2 Department of Pharmacology, School of Basic Medicine, Nanjing Medical University, Nanjing, 210000 China; 3 Department of Anesthesiology, Tongji Hospital, Tongji Medical College, Huazhong University of Science and Technology, Wuhan, 430030 China; 4 Department of Central Laboratory, Affiliated Hospital of Jiangsu University, Zhenjiang, 212000 China; 5 Department of Anesthesiology and Perioperative Medicine, The University of Texas MD Anderson Cancer Center, Houston, TX 77030 USA

**Keywords:** Inflammatory pain, AMPK, IL-1β, NF-κB

## Abstract

**Background:**

Chronic pain is a major clinical problem with limited treatment options. Previous studies have demonstrated that activation of adenosine monophosphate-activated protein kinase (AMPK) can attenuate neuropathic pain. Inflammation/immune response at the site of complete Freund’s adjuvant (CFA) injection is known to be a critical trigger of the pathological changes that produce inflammatory pain. However, whether activation of AMPK produces an analgesic effect through inhibiting the proinflammatory cytokines, including interleukin-1β (IL-1β), in inflammatory pain remains unknown.

**Methods:**

Inflammatory pain was induced in mice injected with CFA. The effects of AICAR (5-aminoimidazole-4-carboxyamide ribonucleoside, an AMPK activator), Compound C (an AMPK inhibitor), and IL-1ra (an IL-1 receptor antagonist) were tested at day 4 after CFA injection. Inflammatory pain was assessed with von Frey filaments and hot plate. Immunoblotting, hematoxylin and eosin (H&E) staining, and immunofluorescence were used to assess inflammation-induced biochemical changes.

**Results:**

The AMPK activator AICAR produced an analgesic effect and inhibited the level of proinflammatory cytokine IL-1β in the inflamed skin in mice. Moreover, activation of AMPK suppressed CFA-induced NF-κB p65 translocation from the cytosol to the nucleus in activated macrophages (CD68^+^ and CX3CR1^+^) of inflamed skin tissues. Subcutaneous injection of IL-1ra attenuated CFA-induced inflammatory pain. The AMPK inhibitor Compound C and AMPKα shRNA reversed the analgesic effect of AICAR and the effects of AICAR on IL-1β and NF-κB activation in inflamed skin tissues.

**Conclusions:**

Our study provides new information that AMPK activation produces the analgesic effect by inhibiting NF-κB activation and reducing the expression of IL-1β in inflammatory pain.

**Electronic supplementary material:**

The online version of this article (10.1186/s12974-019-1411-x) contains supplementary material, which is available to authorized users.

## Background

Noxious stimuli to peripheral tissues result in pain hypersensitivity, as a result of peripheral sensitization of primary sensory neurons [1, 2]. Complete Freund’s adjuvant (CFA) induces cell-mediated immunity and potentiates the production of immunoglobulins as an immunopotentiator [3, 4]. These reactions induced by CFA lead to the tissue inflammation at the injection site and the release of cytokines. Adenosine monophosphate-activated protein kinase (AMPK) is an enzyme of cellular energy homeostasis as a metabolic stress-sensing protein [5, 6]. Phosphorylation of AMPK acts as a positive regulator of other pathways, such as AMPK/SIRT1 and AMPK/mTOR, and has an analgesic effect on neuropathic pain [7, 8]. Activation of AMPK and the downstream protein p65 NF-κB are critically involved in controlling neuroinflammation [9] and inflammatory arthritis [10]. However, the role of AMPK/NF-κB in inflammatory pain is unclear.

The enhanced cannabinoid type 2 (CB2) receptor activation inhibits Nod-like receptor proteins-3 (NLRP3) inflammasomes to reduce the release of interleukin-1β (IL-1β) p17, a mature fragment of IL-1β, which contributes to CFA-induced pain hypersensitivity [11, 12]. Proinflammatory cytokines, such as tumor necrosis factor α (TNFα) and IL-1β, are important contributors in pain signaling [13, 14]. IL-1β is expressed by activated macrophages as proprotein and is proteolytically processed to its mature fragment by Caspase 1. The mature fragment of IL-1β causes sensitization of nerve fibers of primary sensory neurons to cause pain hypersensitivity [15, 16]. Whether activation of AMPK inhibits CFA-induced upregulation of IL-1β is unclear. In this study, we tested a hypothesis that AMPK activation decreases inflammatory pain via inhibiting NF-κB activation and downregulating IL-1β.

Since there was no report that activation of AMPK exerts its anti-inflammatory in mice of CFA-induced inflammatory pain model, we employed AICAR, an AMPK activator; Compound C, an AMPK inhibitor; and recombination mouse IL-1ra, a receptor antagonist of IL-1β, to explore the relationship between AMPK, p65 NF-κB, and IL-1β.

## Methods

### Animal models

All procedures were strictly carried out in accordance with the ethical guidelines of the International Association for the Study of Pain [17]. Male C57BL/6 mice (8–9 weeks old) were purchased from Beijing Vital River Laboratory Animal Technology Co., Ltd. All animals were housed four to six per cage on a cycle of 12 h light and 12 h dark (temperature, 22–24 °C) and had free access to water and food. They were allowed to adapt to these conditions for at least 3 days before all experiments. Inflammatory pain model was induced by CFA (25 μl, subcutaneously injection) on the dorsolateral surface (hairy skin) of the left hindpaw, as previously reported [10]. Control mice were injected with 25 μl of saline to the left hindpaw. The B6.129P-Cx3cr1tm1Litt/J mice (CX3CR1-GFP mice, The Jackson Laboratory, USA) express EGFP in monocytes. CX3CR1 is one of the markers of macrophages [18]. CX3CR1-GFP mice were used for the same experiments as described for C57BL/6 wild-type mice. An additional file shows the experimental design timeline in more detail (see Additional file 1).

### Drugs and reagents

Complete Freund’s adjuvant (CFA) (#F5881) was purchased from Sigma-Aldrich. AICAR (#9944) was obtained from Cell Signaling Technology, and Compound C (# ab120843) was purchased from Abcam. Recombinant mouse IL-1ra/IL-1F3 (# 480-RM-050) was purchased from R&D systems.

### Assessment of peripheral CFA-induced inflammatory pain behaviors

All mice were adapted to the testing environment for 30 min before testing the baseline nociceptive thresholds. The baseline nociceptive thresholds were tested for three successive days prior to CFA injection, and the thresholds on the third day were calculated as the baseline. After CFA injection, animal behavior tests were performed once a day. To examine the effect of drugs and vehicles on the inflammatory pain, tests were conducted daily within 1–4 days and at different points of time after a single drug injection at day 4 after CFA injection.

The tactile withdrawal threshold of mice was measured by using the “up-down” method [19]. After an acclimation period of 30 min, we stimulated the plantar surface of the left hindpaw vertically with a series of von Frey filaments (Stoelting, Wood Dale, IL) with logarithmically increasing stiffness. The filament was bent for 5 s to the central plantar surface with a sufficient force, and brisk withdrawal or paw flinching was considered as a positive response. The test of tactile withdrawal threshold was repeated two times in each mouse, and the mean value was calculated.

The thermal pain threshold of mice was tested by using a hot plate. The surface temperature of the hot plate was maintained at 53 °C. The withdrawal latency started from putting the mouse on the plate and terminated when a quick withdrawal or flick of the paw was observed. Twenty seconds was set as a cutoff time to prevent tissue damage [20]. Thermal tests were repeated three times at 10-min intervals, and the mean value was calculated.

### H&E staining

Mice were deeply anesthetized with 10% chloralic hydras and were transcardially perfused with 37 °C normal saline followed by 4% paraformaldehyde in 0.1 M PBS (pH, 7.4; 4 °C). Then, the tissues were removed immediately and fixed in 10% buffered formalin for 48 h and prepared for routine paraffin histology. The paraffin-embedded 5-μm-thick sections from different groups were stained by hematoxylin and eosin (H&E).

### Western blotting

In brief, mice were deeply anesthetized with 10% chloralic hydras, and their skin tissues were removed immediately. The tissues were minced with scissors and then lysed by adding RIPA Lysis Buffer (10 μl/mg for tissue, Beyotime Biotechnology) with protease and phosphatase inhibitor cocktail (1 mM, Guge Biotech, Wuhan, China) for 30 min. Then, tissue lysates were centrifuged at 12,000 rpm 4 °C for 15 min, and the protein contents were quantified by using the BCA Protein Assay Kit (Dingguo Changsheng Biotechnology, Beijing, China). Proteins (60 μg) were separated by SDS-PAGE and transferred onto PVDF membranes (Millipore Corp.). The membranes were incubated with 5% bovine serum albumin (BSA) for 1 h at room temperature. Then, the membranes were probed with primary antibodies overnight at 4 °C. The primary antibodies included p-AMPK (Thr172) (Cell Signaling Technology, #2535), 1:1000; AMPK (Cell Signaling Technology, #2532), 1:1000; IL-1β (R&D Systems, #MAB5011), 1:1000; CD68 (Proteintech, #28058-1-AP), 1:1000; GFP (Santa Cruz Biotechnology, sc-9996), 1:1000; and β-actin (Santa Cruz Biotechnology, sc-47778), 1:10000. The signals were developed using Pierce ECL Western Blotting Substrate (Thermo Fisher Scientific, USA). Densitometry analysis of the gel images was performed using the ImageJ software (NIH, Bethesda, MD, USA).

### Immunofluorescence

Mice under deep anesthesia with 10% chloralic hydras were transcardially perfused with 37 °C normal saline followed by 4% paraformaldehyde in 0.1 M PBS (pH, 7.4; 4 °C). The skin tissues at inflammatory sites (left hindpaw), on the ipsilateral side of CFA injection, were removed immediately and post-fixed in the same fixative. Then, the tissues were cryoprotected in 20% and 30% sucrose in 0.1 M PBS for 24 h at 4 °C. The OCT-embedded blocks were sectioned at 20 μm thickness. Sections from each group were rinsed in 0.01 M PBS and blocked for 2 h with blocking solution (5% donkey serum and 0.2% tween-20 in 0.01 M PBS) at room temperature. The sections were probed with the following antibodies: rabbit anti-NF-κB p65 (Abcam, #ab90532) (1:200), rabbit anti-p-AMPK (Abcam, #ab23875) (1:250), and mouse anti-CD68 (Abcam, #ab955) (1:200) (marker of macrophages [21]). Subsequently, the free-floating sections were washed with 0.01 M PBS for three times and incubated with the following secondary antibodies (Jackson ImmunoResearch, USA) for 2 h: donkey anti-rabbit IgG conjugated with Dylight 594 (1:600) and donkey anti-mouse IgG conjugated with Dylight 488 or Dylight 594 (1:600). The sections were incubated with diamidino-phenyl-indole (DAPI) for the nucleus staining for 8 min, washed three times in 0.01 M PBS, and then cover-slipped. The sections were examined under a fluorescence microscope (Olympus BX51) for the immunofluorescence staining. Images were analyzed by using NIH ImageJ software (Bethesda, MD, USA).

### shRNA-mediated knockdown of AMPKα

To decrease the expression of AMPKα1 and AMPKα2, AMPKα1/2 shRNA(m) Lentiviral Particles (Santa Cruz Biotechnology, USA) were used to inhibit the expression of AMPKα1 and AMPKα2. On the second day after CFA injection, 15 μl shRNA was subcutaneously injected into the sites of inflammation. The dose of shRNA Lentiviral Particles was selected based on our preliminary experiments, and the shRNA selected was the most effective in reducing AMPKα expression.

### Statistical analyses

Results are expressed as mean ± SEM. The thermal latency and withdrawal thresholds between different groups over time were tested with two-way analysis of variance (ANOVA) followed by Bonferroni post hoc tests. One-way ANOVA and Newman-Keuls post hoc test were used to compare protein expression levels (SPSS, version 11.0). A *P* value of < 0.05 was considered to be statistically significant. Significance between two groups was evaluated using non-paired Student’s *t* test.

## Results

### Subcutaneous injection of AICAR alleviated CFA-induced pain hypersensitivity and upregulated phosphorylated AMPK in inflamed skin tissues of mice

**Fig. 1:**
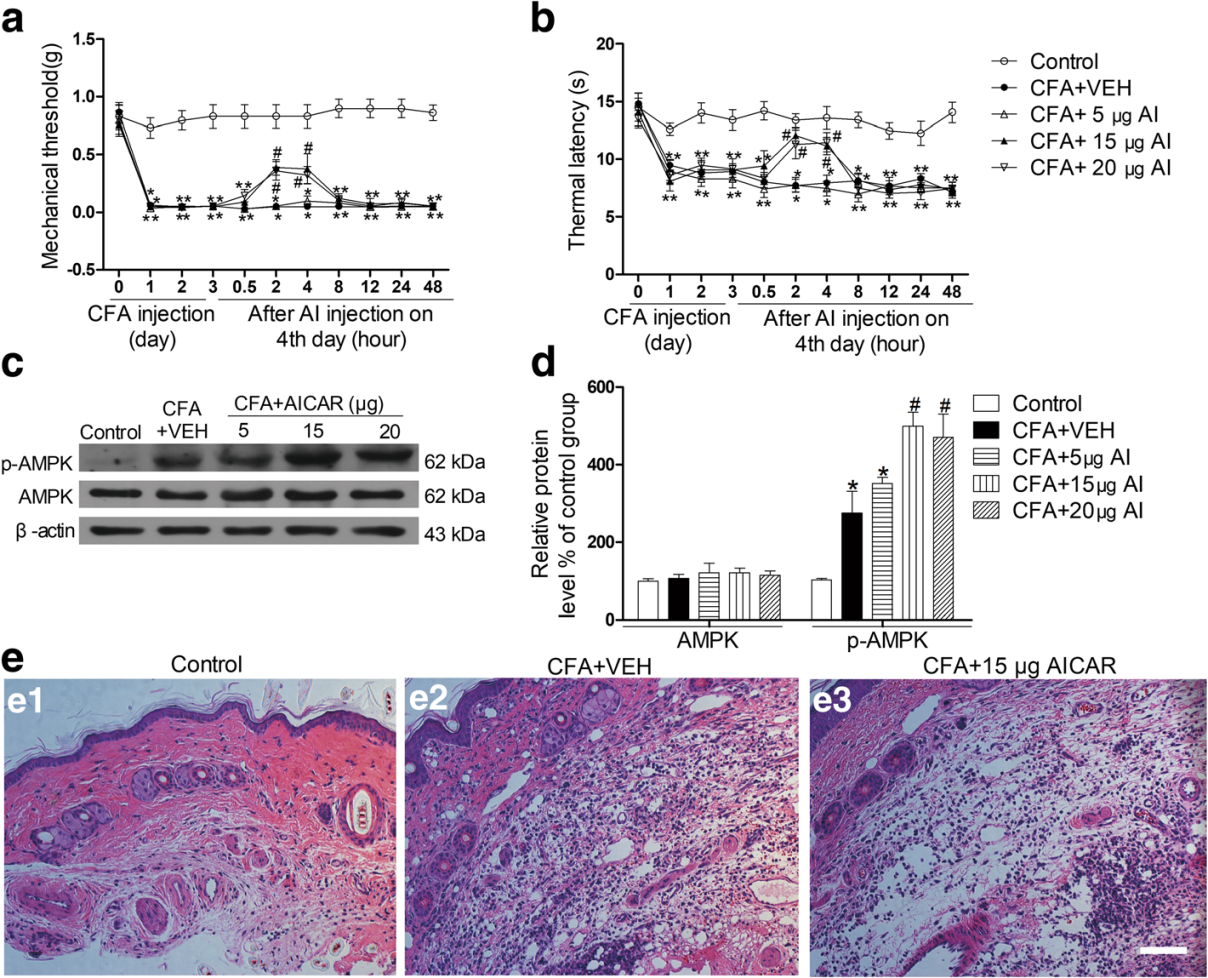
AICAR attenuates CFA-induced pain behavior and local inflammation and promotes phosphorylation of AMPK in mice. Effects of a single hypodermic injection of AICAR (5, 15, or 20 μg/20 μl) on mechanical allodynia (**a**) and thermal hyperalgesia (**b**) in CFA-injected mice. The panels show the hindpaw withdrawal threshold in response to mechanical stimulation as assessed with von Frey hairs in **a** or thermal stimulation as assessed with hot plate in **b** (data are expressed as mean ± SEM, *n* = 8 mice/group). Two-way ANOVA revealed a significant difference at *P < 0.05 vs. Control group and ^#^*P* < 0.05 vs. CFA+VEH group. **c**, **d** Subcutaneous administration of AICAR (5, 15, or 20 μg /20 μl) activated AMPK in the inflamed skin tissues in a dose-dependent manner. One-way ANOVA revealed a significant difference at *P < 0.05 vs. Control group and ^#^*P* < 0.05 vs. CFA+VEH group. **e** H&E staining. (e1) Control mice with no inflammation in the skin, (e2) inflammation with abundant lymphocytes and sparse neutrophilic granulocytes in an CFA mouse with hypodermic injection of vehicle (20 μl sterilized PBS, the vehicle of AICAR), and (e3) low-grade inflammation in an CFA mouse with hypodermic injection of AICAR (15 μg/20 μl). Scale bar represents 100 μm. Tissues were collected at 2 h after AICAR treatment for Western blotting and HE. The abbreviations used here are VEH (vehicle of AICAR, sterilized double steamed H_2_O) and AI (AICAR)

The induction/acute phase of CFA-induced inflammatory pain usually takes about 2 days, and then, CFA-induced inflammatory pain becomes persistent, lasting at least for several weeks [22, 23]. For this reason, we conducted our experiments at day 4 after CFA injection. All mice that received CFA injection exhibited mechanical allodynia and thermal hyperalgesia. A single subcutaneous administration of AICAR (5 μg, 15 μg, and 20 μg in 20 μl for each single injection) at day 4 after CFA injection significantly suppressed mechanical allodynia and thermal hyperalgesia in a dose-dependent manner, and the analgesic effects of AICAR lasted for > 4 h (Fig. 1a, b). Two hours of AICAR treatment also markedly promoted the phosphorylation of AMPK in a dose-dependent manner (Fig. 1c, d). In addition, the control mice displayed no inflammation (Fig. 1 e1), versus notable inflammatory changes in the CFA-injected mice (Fig. 1 e2). Compared to CFA-injected mice, tissues from CFA + AICAR (15 μg/20 μl) mice showed low-grade inflammation (Fig. 1 e3).

### AMPK activation with AICAR suppressed CFA-induced NF-κB activation in macrophages of inflamed skin tissues and inhibited the expression of pro-IL-1β and IL-1β p17

**Fig. 2:**
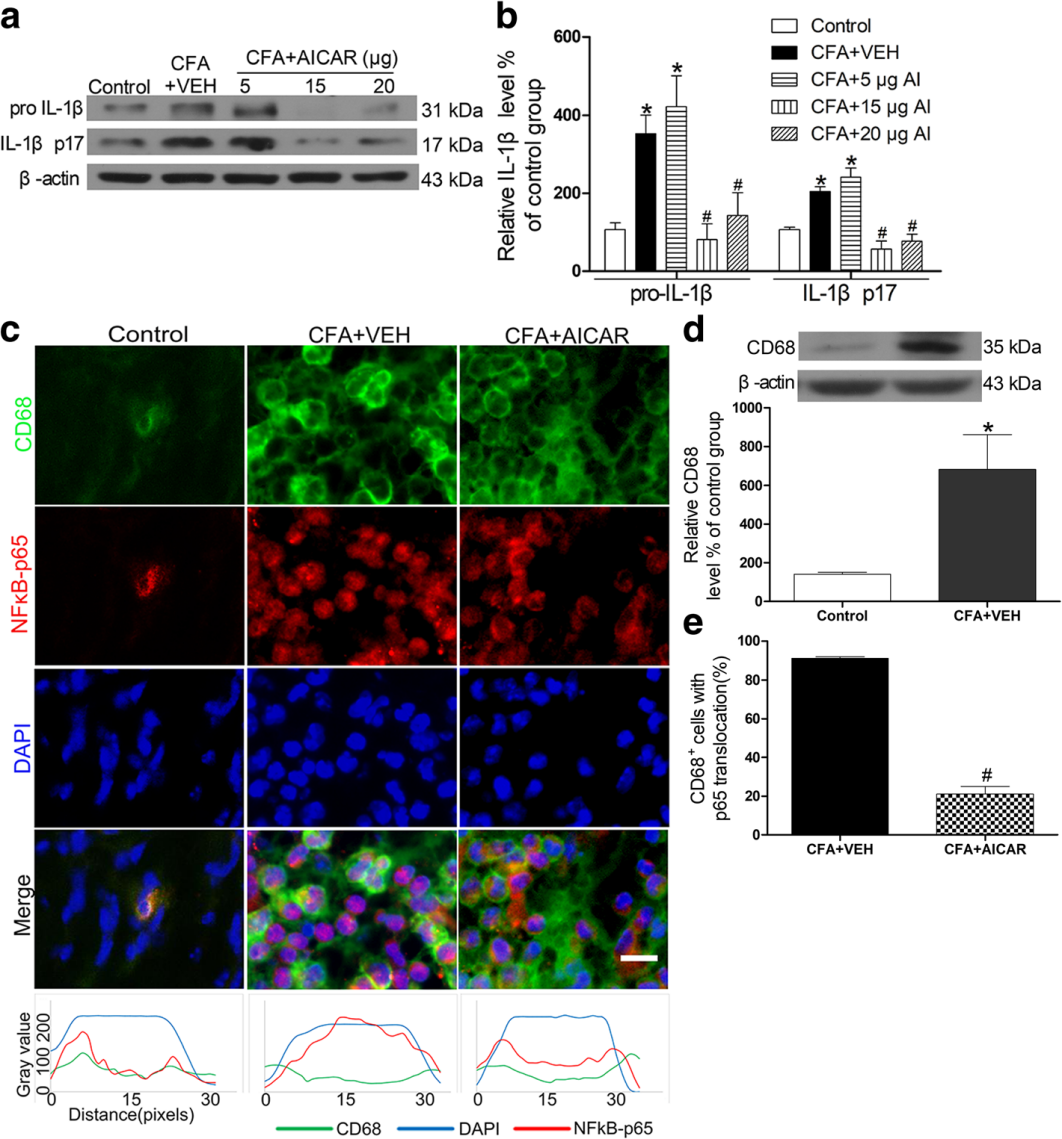
AICAR downregulates expression of IL-1β and suppresses CFA-induced NF-κB activation in macrophages of the inflamed skin. AICAR (5, 15, 20 μg/20 μl) was administrated at CFA injection day 4. Skin tissues were collected at 2 h after AICAR treatment for Western blotting and immunofluorescence. Representative gels and quantification for pro-IL-1β and IL-1β p17 bands (**a** and **b**) are shown. One-way ANOVA revealed a significant difference at ^*^P < 0.05 vs. Control group and ^#^*P* < 0.01 vs. CFA+VEH group. **c** AICAR inhibited the CFA-induced NF-κB translocation from the cytosol to the nucleus, as demonstrated by the immunofluorescence of p65 in CD68 marked macrophage cells. Scale bar represents 50 μm. The bottom line of the panels shows the merged profiles of the fluorescent intensity of CD68, NF-κB p65 and DAPI signals along the lines drawn through the axis of immunostaining macrophage cells. **d** Quantification for CD68 protein level of skin tissues. **e** Quantification for cells with p65 translocation of **c**. Non-paired Student’s *t* test revealed a significant difference at *P < 0.05 vs. Control group and ^#^*P* < 0.01 vs. CFA + VEH group (*n* = 4 mice/group). The abbreviations used here are VEH (vehicle of AICAR) and AI (AICAR)

Upregulated IL-1β p17 in inflamed skin tissues of CFA-induced inflammation is involved in pain hypersensitivity [24]. The upregulated phosphorylation of AMPK has an analgesic effect on neuropathic pain [25]. CFA caused significant increases in the levels of pro-IL-1β and IL-1β p17 (Fig. 2a, b), which occurred at the same time when inflammatory pain developed in mice. Treatment with AICAR (5, 15, and 20 μg/20 μl) by a single subcutaneous injection at day 4 after CFA injection significantly inhibited the expression levels of pro-IL-1β and IL-1β p17 in a dose-dependent manner (Fig. 2a, b).

**Fig. 3:**
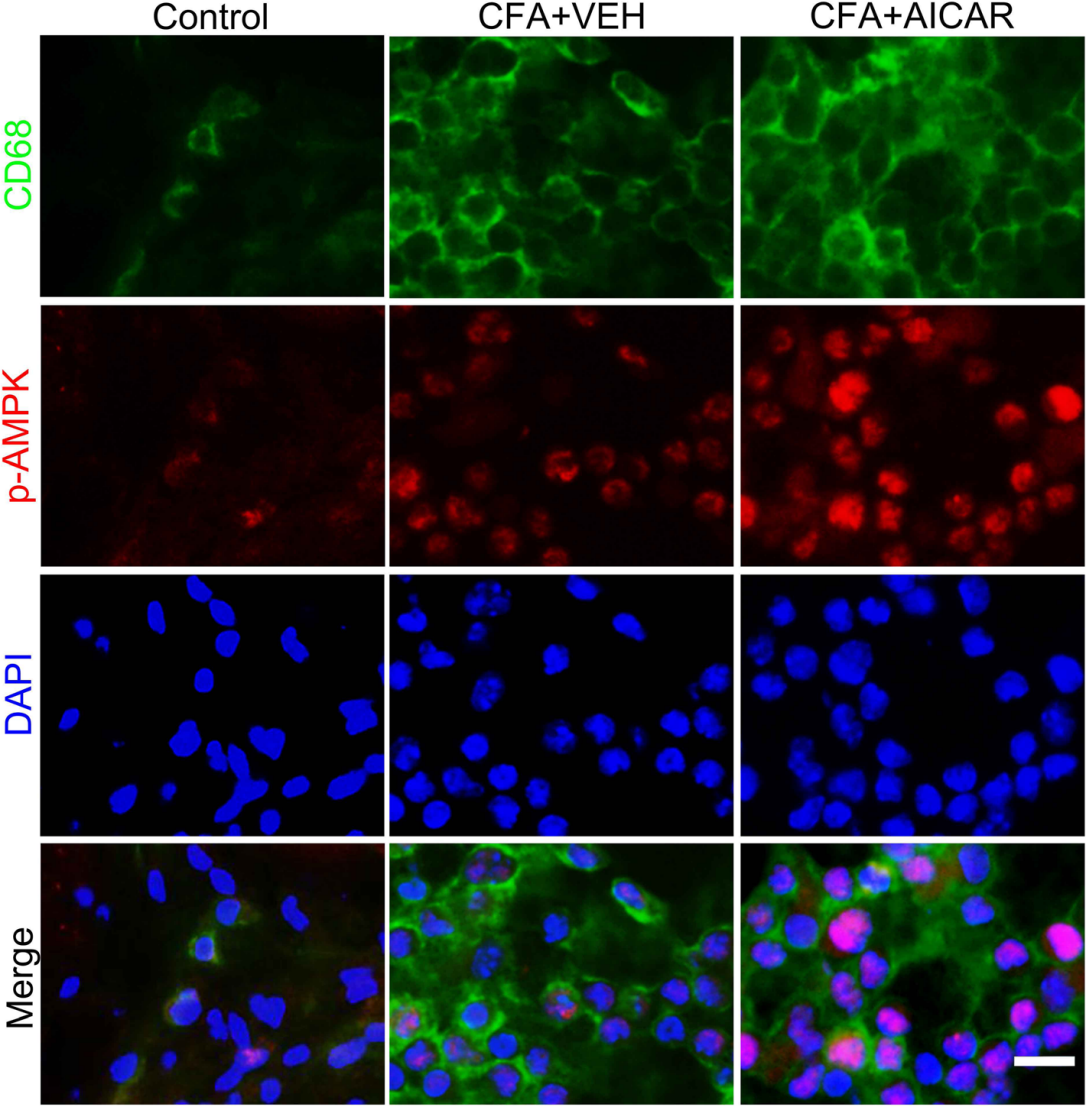
Co-localization of CD68^+^ cells and p-AMPK at day 4 after CFA injection in mice. Immunostaining of skin sections showing the co-localization of CD68^+^ cells and p-AMPK in inflamed skins at day 4 after AICAR administration. Scale bar represents 50 μm. Skin tissues were collected at 2 h after AICAR treatment for immunofluorescence

IL-1β is mainly released from macrophages in inflammation [26]. Proinflammatory cytokines, such as TNFα and IL-1β, contribute to inflammatory pain [11, 12]. Since p65 NF-κB translocation from the cytosol to the nucleus can promote the expression of proinflammatory cytokines in inflammation [26], we determined whether AICAR inhibits the expression of pro-IL-1β by regulating NF-κB activation. CFA increased p65 NF-κB translocation from the cytosol to the nucleus in CD68 marked macrophages (Fig. 2c). This effect was significantly inhibited by treatment with AICAR (Fig. 2c–e). The co-localization of macrophages (CD68^+^) and p-AMPK was identified by immunostaining in inflamed skin tissues (Fig. 3).

### Subcutaneous injection of IL-1ra attenuates CFA-induced pain of mice

**Fig. 4:**
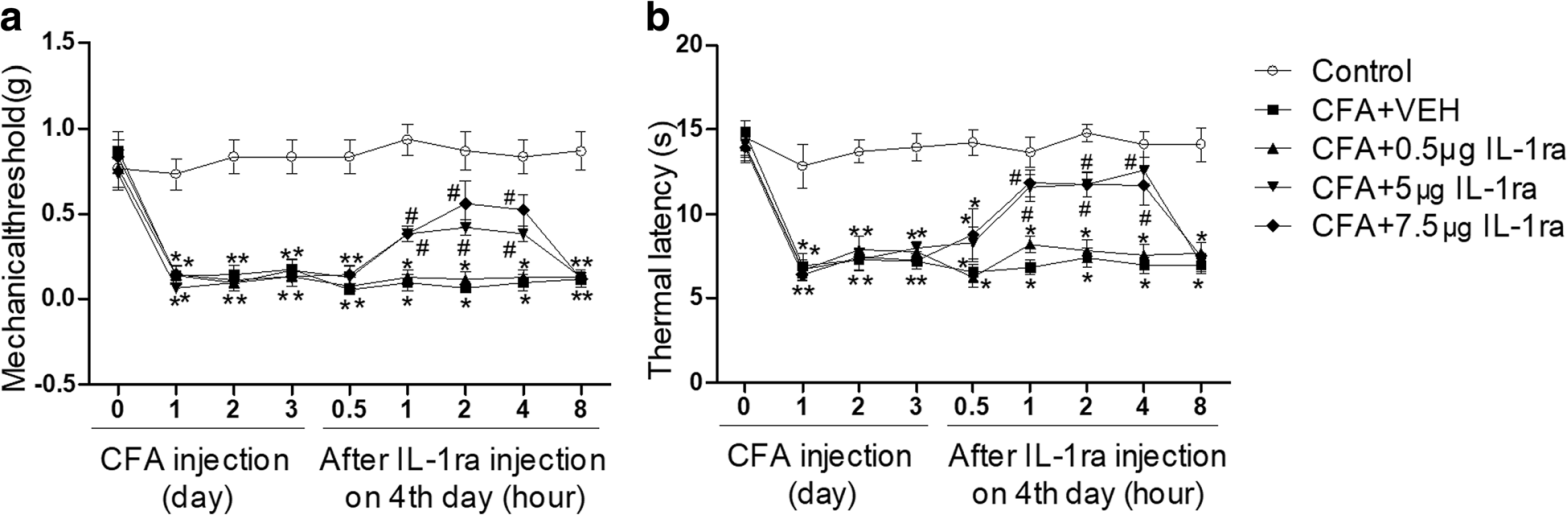
Subcutaneous injection of IL-1ra attenuates CFA-induced inflammatory pain of mice. **a** Effects of a single injection of IL-1ra (0.5, 5, or 7.5 μg/20 μl) on mechanical allodynia induced by CFA in mice. **b** Effects of a single injection of IL-1ra (0.5, 5, or 7.5 μg/20 μl) on thermal hyperalgesia induced by CFA in mice. Data are expressed as mean ± SEM, *n* = 8 mice/group. Two-way ANOVA revealed a significant difference at ^*^P < 0.05 vs. Control group and ^#^*P* < 0.05 vs. CFA + VEH (vehicle of IL-1ra, sterilized PBS) group. AI, AICAR

Since AICAR alleviates CFA-induced inflammatory pain and downregulates the overexpression of IL-1β simultaneously, we determined whether IL-1ra attenuates CFA-induced inflammatory pain. A single subcutaneous administration of IL-1ra (0.5 μg, 5 μg, and 7.5 μg of IL-1ra in 20 μl for each single injection) significantly reduced pain hypersensitivity at day 4 after CFA injection in a dose-dependent manner (Fig. 4a, b).

### AMPK activation is required for the analgesic effect of AICAR on CFA-induced inflammatory pain in mice

**Fig. 5:**
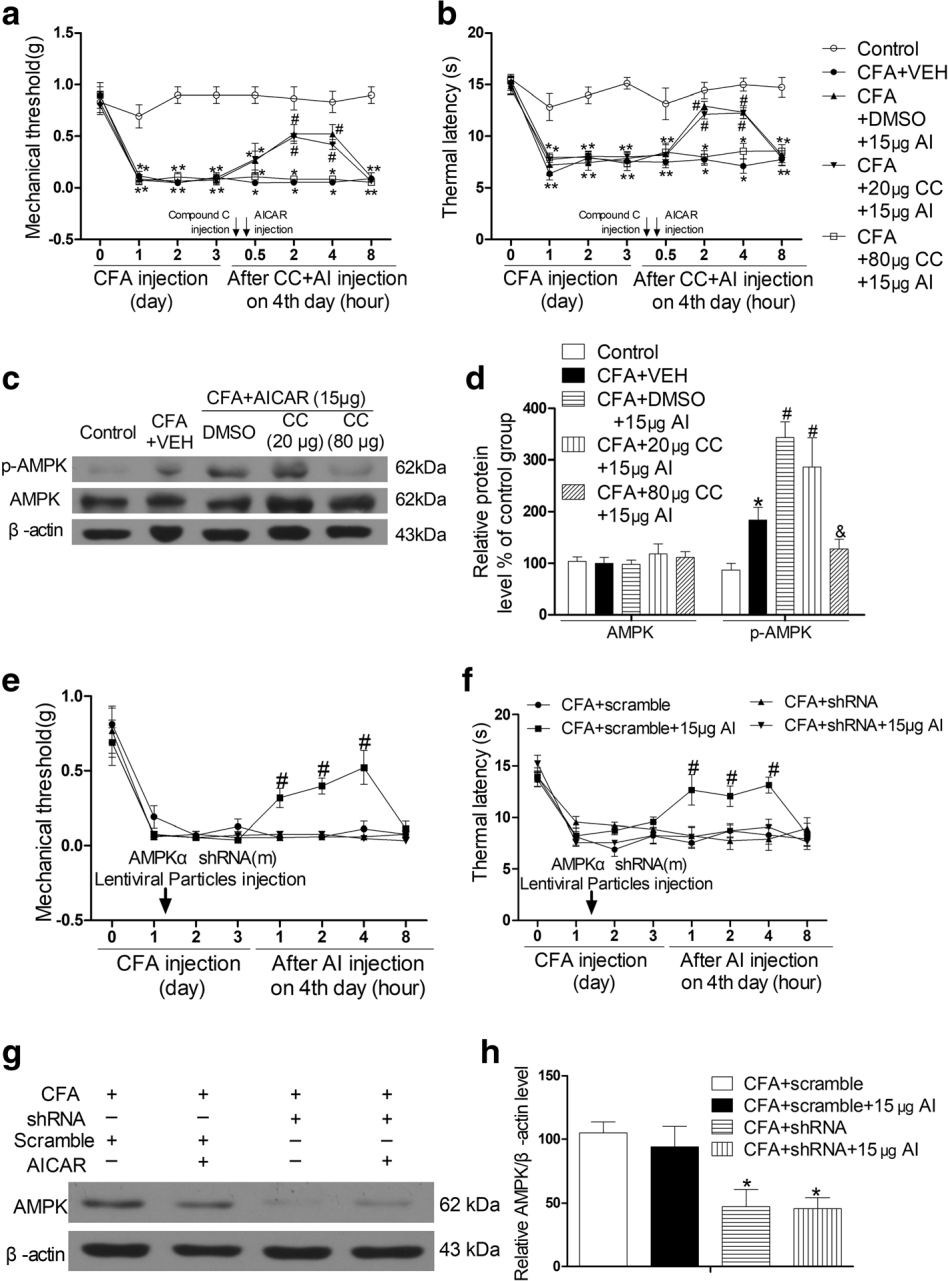
AMPK activation is required for AICAR to attenuate CFA-induced inflammatory pain in mice. Compound C (20 μg, 80 μg/20 μl) was administrated at post-injection of CFA day 4 before AICAR (15 μg/20 μl) treatment for 30 min. **a**–**d** AMPK inhibitor Compound C (80 μg/20 μl) reversed the effects of AICAR (15 μg/20 μl). Effects of a single injection of Compound C (20 μg or 80 μg/20 μl) on mechanical allodynia (**a**) and thermal hyperalgesia (**b**) in CFA-injected mice with 15 μg/20 μl AICAR administration. Two-way ANOVA revealed a significant difference at ^*^P < 0.05 vs. Control group and ^#^*P* < 0.05 vs. CFA+VEH group (*n* = 8 each group). Skin tissues were collected at 2 h after AICAR treatment for Western blotting. Representative gels and quantification for p-AMPK and AMPK bands are shown (**c**, **d**). One-way ANOVA revealed a significant difference at ^*^P < 0.05 vs. Control group and ^#^*P* < 0.05 vs. CFA+VEH group. **e**–**h** Effects of AMPKα shRNA on CFA-induced mechanical allodynia, thermal hyperalgesia, and AICAR (15 μg/20 μl) treatment in mice (*n* = 5 mice/group). After the local administration of AMPKα shRNA, AICAR could not attenuate mechanical allodynia (**e**) and thermal hyperalgesia (**f**) and downregulated the level of AMPKα in inflamed tissues (**g**, **h**). Two-way ANOVA revealed a significant difference at ^#^*P* < 0.05 vs. CFA + shRNA + 15μg AI group (**e** and **f**). One-way ANOVA revealed a significant difference at ^*^P < 0.05 vs. CFA +scramble group (**h**). The abbreviations used here are VEH (vehicle of AICAR) and CC (Compound C, dissolve in DMSO (dimethyl sulphoxide))

The AICAR effects were inhibited by the AMPK inhibitor Compound C (80 but not 20 μg, subcutaneous injection) (Fig. 5a, b). Also, 80 μg Compound C significantly inhibited AICAR-induced phosphorylation of AMPK in inflamed skin tissues of CFA-injected mice (Fig. 5c, d). Subcutaneous injection of AMPKα-specific shRNA also abolished AICAR-mediated suppression of mechanical allodynia and thermal hyperalgesia in CFA-injected mice (Fig. 5e, f). RNA interference of expression of AMPKα was confirmed by Western blotting (Fig. 5g, h). These data suggest that AICAR reduces inflammatory pain by activating AMPK.

### AMPK activation is required for AICAR to downregulate the CFA-induced increase of IL-1β in inflamed skin tissues

**Fig. 6:**
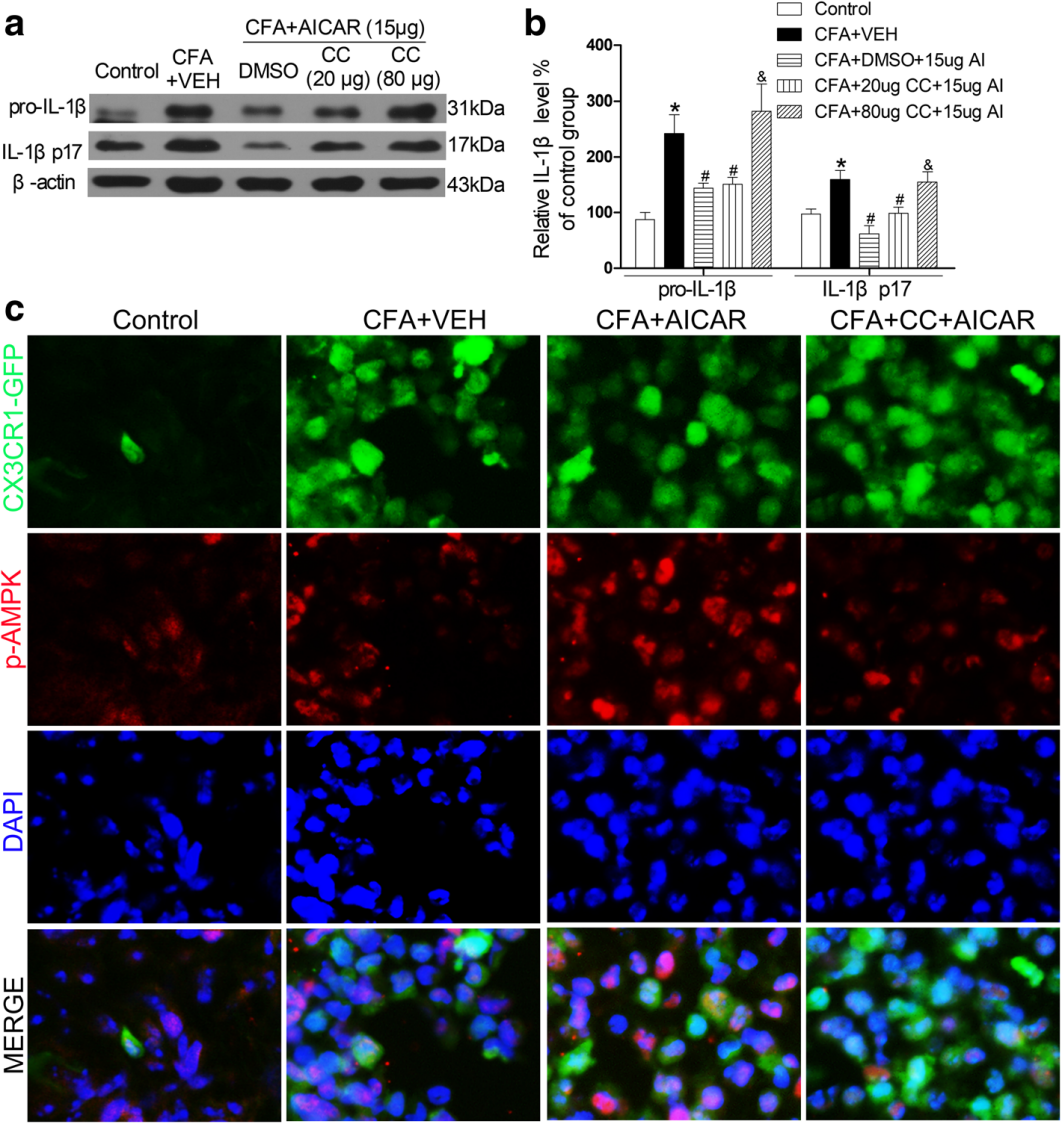
AMPK inhibitor Compound C reverses the effects of AICAR. Compound C (20 μg, 80 μg/20 μl) was administrated at post-injection of CFA day 4 before AICAR (15 μg/20 μl) treatment 30 min. Skin tissues were collected at 2 h after AICAR treatment. Representative gels and quantification for pro-IL-1β and IL-1β p17 bands are shown (**a**, **b**). Data are expressed as mean ± SEM. One-way ANOVA revealed a significant difference at ^*^P < 0.05 vs. Control group and ^#^*P* < 0.05 vs. CFA+VEH group (*n* = 4 mice/group). The abbreviations used here are VEH (vehicle of AICAR), AI (AICAR) and CC (Compound C). **c** Immunostaining of skin sections showing the co-localization of CX3CR1-GFP ^+^ cells and p-AMPK in inflamed skins on the fourth day (**c**). Scale bar represents 50 μm

Next, we determined whether the AMPK inhibitor Compound C reverses the inhibitory effects of AICAR on the increased expression levels of IL-1β in CFA-injected mice. Indeed, subcutaneous injection of AICAR (15 μg/20 μl) markedly inhibited the expression level of pro-IL-1β and IL-1β p17; these effects were reversed by Compound C significantly (80 μg/20 μl) (Fig. 6a, b). Together with the results of IL-1ra, AMPK activation attenuates the CFA-induced pain via reducing IL-1β.

### AMPK activation is required for AICAR to suppress CFA-induced NF-κB activation in macrophages of inflamed skin tissues

**Fig. 7:**
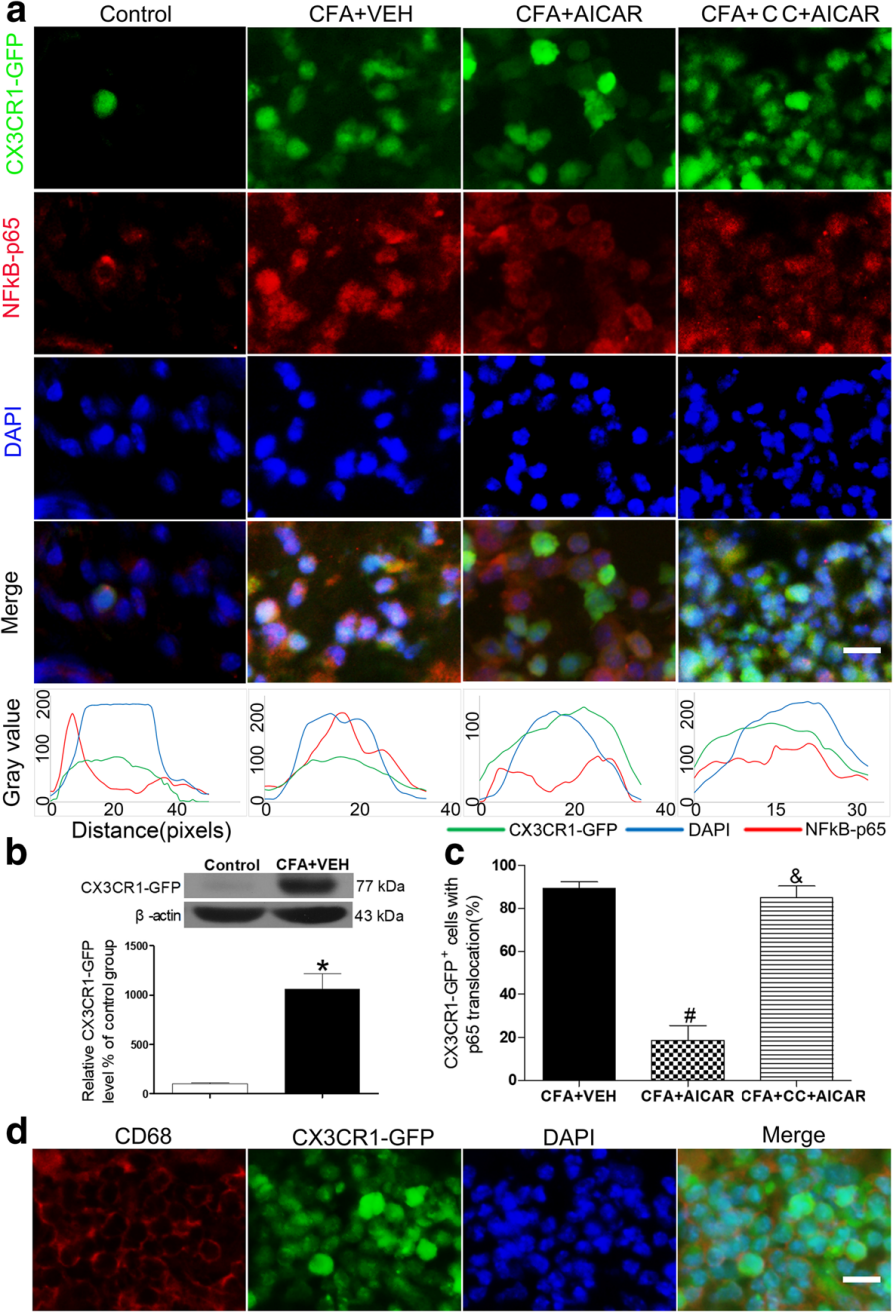
AMPK activation is required for inhibiting NF-κB p65 translocation. Compound C reverses the effects of AICAR to suppress CFA-induced NF-κB p65 translocation in CX3CR1-GFP mice. After a pre-treatment of Compound C (80 μg/20 μl), mice were treated with AICAR (15 μg/20 μl). Skin tissues from CX3CR1-GFP mice were collected at 2 h after AICAR treatment for immunofluorescence. **a** AICAR inhibited the CFA-induced NF-κB translocation from the cytosol to the nucleus, as demonstrated by the immunofluorescence of NF-κB p65 in CX3CR1-GFP-marked macrophage cells. Scale bars represent 50 μm. The same experimental results were repeated in four mice of each group. The bottom line of the panels shows the merged profiles of the fluorescent intensity of CX3CR1-GFP, NF-κB p65, and DAPI signals along the lines drawn through the axis of CX3CR1-GFP-positive macrophage cells. **b** Quantification for CX3CR1-GFP protein level of skin tissues. **c** Quantification for CX3CR1-GFP macrophages with p65 translocation of **a**. Non-paired Student’s *t* test revealed a significant difference at *P < 0.05 vs. Control group, ^#^*P* < 0.01 vs. CFA + VEH group, and ^&^*P* < 0.05 vs. CFA + AICAR group, (*n* = 4 mice/group). **d** The panels are images showing double-labeled cells in CX3CR1-GFP mice (after CFA injection at day 4) with CD68 and DAPI. The abbreviations used here are VEH (vehicle of AICAR) and CC (Compound C)

Treatment of AICAR inhibited p65 NF-κB translocation from the cytosol to the nucleus in CD68-marked macrophages induced by CFA in inflamed skin tissues (Fig. 2c), and co-localization of CX3CR1-GFP ^+^ cells (macrophages) and p-AMPK was identified by immunostaining in inflamed skins (Fig. 6c). We determined whether the AMPK activator AICAR inhibits the p65 NF-κB translocation in CX3CR1-GFP mice. Treatment of AICAR (15 μg/20 μl) inhibited p65 NF-κB translocation from the cytosol to the nucleus in CX3CR1-GFP cells, which was blocked by Compound C significantly (80 μg/20 μl, 30 min before AICAR injection) (Fig. 7a–c). In addition, double immunofluorescence labeling confirmed the coexistence of CD68 and CX3CR1-GFP in inflamed skin tissues 4 days after CFA injection (Fig. 7d). Since p65 NF-κB activation promotes the expression of proinflammatory cytokines in inflammation [27], AMPK activation reduces CFA-induced overexpression of IL-1β and pain hypersensitivity by inhibiting p65 NF-κB translocation from the cytosol to the nucleus in activated macrophages.

## Discussion

Alteration of proinflammatory cytokines in the tissues plays a crucial role in alleviating painful conditions [11, 28]. Activation of AMPK exerts analgesic effects through various pathways in many pain models, such as chronic constriction injury (CCI)-induced neuropathic pain [25], incision-evoked pain [29], and painful diabetic neuropathy [30]. In the present study, we found that AMPK activator AICAR exerted an analgesic effect and inhibited the proinflammatory cytokine IL-1β and IL-1β p17 in mice of CFA-induced inflammatory pain. Specifically, activation of AMPK suppressed CFA-induced NF-κB p65 translocation from the cytosol to the nucleus in activated macrophages and inhibited the expression of IL-1β in inflamed skin tissues. The AMPK inhibitor Compound C and AMPKα shRNA reversed the analgesic effect of AICAR and inhibited the effect of AICAR on downregulating CFA-induced increase of IL-1β and suppressing NF-κB activation in inflamed skin tissues. Our study demonstrated for the first time that AMPK activation plays an important role in the analgesic effect of AICAR by inhibiting NF-κB activation and IL-1β overexpression in CFA-induced inflammatory pain.

AMPK is a common kinase regulated by a variety of pharmacological entities [31] and has three subunits of a catalytic α and regulatory β and γ [32]. Phosphorylation of AMPKα at Thr172 is required for AMPK activation [33, 34]. In addition, activation of AMPK had a strong effect on anti-inflammatory in animal models of diabetic neuropathy [35], arthritis [10], and severity of acute lung injury [36]. In our study, AICAR significantly increased phosphorylation of AMPK, inhibited overexpression of proinflammatory cytokine IL-1β, and attenuated the CFA-induced pain hypersensitivity. Treatment with AMPK inhibitor blocked the effect of AICAR in CFA-injected mice, showing that activation of AMPK played an important role in analgesic effect on CFA-induced inflammatory pain.

CFA induces proinflammatory cytokine secretion in arthritis, and lipopolysaccharide (LPS) induced NF-kB p65 translocation from the cytosol to the nucleus in RAW 264.7 cells (a macrophage cell line) [37]. Inflammation is associated with the activation of the NF-κB signaling pathway [38]. NF-kB is a downstream signaling of AMPK [39]. However, whether the AMPK is involved in CFA-induced inflammatory pain by regulating NF-κB activation is unclear. In our study, we found that activation of AMPK blocked NF-kB p65 translocation from the cytosol to the nucleus in activated macrophages of inflamed skin tissues. These results were confirmed in the macrophages of wild-type C57 mice marked with CD68 and CX3CR1-GFP mice. These results were in line with berberine that suppressed LPS-induced overexpression of proinflammatory cytokines including IL-1β through AMPK activation in macrophages in vitro [40]. IL-1β is produced by activated macrophages and upregulated by NF-κB activation in inflammation [27]. These results showed that activation of AMPK inhibited IL-1β secretion from activated macrophages through suppressing NF-κB activation in CFA-induced inflamed skin tissues.

The release of proinflammatory cytokines is an important cause of inflammatory pain. During the inflammatory process, the release of cytokines (e.g., TNF-α, IL-1β, and IL-6) results in nociceptor sensitization [41]. IL-1β is involved in hypersensitivity of many pain models, such as pain in experimental autoimmune encephalomyelitis [42], sciatic pain [43], and CFA-induced inflammatory pain [44]. In the present study, blocking IL-1R reduced pain hypersensitivity of CFA-injected mice. We also confirmed that AMPK activation attenuated inflammatory pain via reducing IL-1β. Although inhibiting IL-1R suppressed CFA-induced inflammatory pain, the detailed molecular mechanisms need further studies.

Our results are consistent with other studies that AMPK activation is involved in analgesia. Metformin can activate AMPK to alleviate neuropathic pain, such as nerve injury-caused neuropathic pain [45] and chemotherapy-induced painful neuropathy [46]. As an activator of AMPK, metformin may be used for treating inflammatory pain conditions. In addition, the upstream signaling of AMPK, cannabinoid receptor 1/2 and κ-opioid receptors, is known to participate in analgesia [47–49], and activation of these receptors promotes AMPK activation [47, 50, 51]. The cannabinoid receptor and opioid receptor agonists have many side effects, such as euphoria and addiction [52, 53]. Thus, AMPK could be a promising analgesic target to treat patients suffering from various painful conditions.

## Conclusions

In conclusion, our findings suggest that activation of AMPK attenuates inflammatory pain by inhibiting the overexpression of IL-1β in inflamed skin tissues through suppressing the NF-kB in activated macrophages. Furthermore, blocking IL-1R of IL-1β is important for alleviating inflammatory pain. Our findings provide novel mechanisms through which AMPK activation reduces inflammatory pain.

## Additional file

Additional file1: **Figure S1.** Experimental design timeline. a. Nociceptive behavioral tests of AICAR effect in mice. Nociceptive behavioral tests were performed before (baseline) and 1 to 4 days after CFA injection. In the CFA plus AICAR group, rats received AICAR administration on 4th day after CFA injection. Ultimate effect of AICAR was measured at 0.5, 2, 4, 8, 12, 24, 48 h after the AICAR treatment. b. Biochemical experiments in mice. At day 4 of CFA injection, after 2 h of AICAR treatment, tissues for Western blotting and immunofluorescence labeling were collected in mice. c. Nociceptive behavioral tests of the effect of IL-1 ra in mice. Nociceptive behavioral tests were performed before (baseline) and 1 to 4 days after CFA injection. Behavior tests of ultimate effects of IL-1ra were performed at 0.5, 1, 2, 4, 8 h after IL-1ra treatment. d. Nociceptive behavioral tests of effects of Compound C on AICAR in CFA injection mice. e. Biochemical experiments in wild-type mice and CX3CR1-GFP mice. At day 4 of CFA injection, after 2 h of AICAR treatment (Compound C administration in the 30 min of AICAR), tissues for Western blotting were collected in wild-type mice, and tissues for immunofluorescence labeling were collected in CX3CR1-GFP mice. f. To determine whether knockdown of AMPKα may reverse AICAR effects, mice were injected with AMPKα shRNA Lentiviral Particles at CFA injection day 1. Behavior tests of ultimate effect of AICAR were performed at 1, 2, 4, 8 h after AICAR treatment at day 4. Tissues for Western blotting were collected after nociceptive behavior tests. Abbreviations: WB, Western blotting; IF, Immunofluorescence labeling. (PDF 8021 kb)

## Abbreviations

AICAR (AI): 5-Aminoimidazole-4-carboxyamide ribonucleoside
AMPK: Monophosphate-activated protein kinase
CC: Compound C
CFA: Complete Freund’s adjuvant
H&E staining: Hematoxylin and eosin staining
IL-1R: Interleukin-1 receptor
IL-1ra: Interleukin-1 receptor antagonist
IL-1β: Interleukin-1beta
NF-κB: Nuclear factor kappa-B
TNF-α: Tumor necrosis factor alpha
VEH: Vehicle
