# Supplementary material for: AMPK activation attenuates inflammatory pain through inhibiting NF-κB activation and IL-1β expression
Source: J Neuroinflammation. 2019 Feb 12;16:34. doi: 10.1186/s12974-019-1411-x (PMC6373126; doi:10.1186/s12974-019-1411-x)
Supplement: Supplementary file 1 — Figure S1. Experimental design timeline. a. Nociceptive behavioral tests of AICAR effect in mice. Nociceptive behavioral tests were performed before (baseline) and 1 to 4 days after CFA injection. In the CFA plus AICAR group, rats received AICAR administration on 4th day after CFA injection. Ultimate effect of AICAR was measured at 0.5, 2, 4, 8, 12, 24, 48 h after the AICAR treatment. b. Biochemical experiments in mice. At day 4 of CFA injection, after 2 h of AICAR treatment, tissues for Western blotting and immunofluorescence labeling were collected in mice. c. Nociceptive behavioral tests of the effect of IL-1 ra in mice. Nociceptive behavioral tests were performed before (baseline) and 1 to 4 days after CFA injection. Behavior tests of ultimate effects of IL-1ra were performed at 0.5, 1, 2, 4, 8 h after IL-1ra treatment. d. Nociceptive behavioral tests of effects of Compound C on AICAR in CFA injection mice. e. Biochemical experiments in wild-type mice and CX3CR1-GFP mice. At day 4 of CFA injection, after 2 h of AICAR treatment (Compound C administration in the 30 min of AICAR), tissues for Western blotting were collected in wild-type mice, and tissues for immunofluorescence labeling were collected in CX3CR1-GFP mice. f. To determine whether knockdown of AMPKα may reverse AICAR effects, mice were injected with AMPKα shRNA Lentiviral Particles at CFA injection day 1. Behavior tests of ultimate effect of AICAR were performed at 1, 2, 4, 8 h after AICAR treatment at day 4. Tissues for Western blotting were collected after nociceptive behavior tests. Abbreviations: WB, Western blotting; IF, Immunofluorescence labeling. (PDF 8021 kb) [file 12974_2019_1411_MOESM1_ESM.pdf]

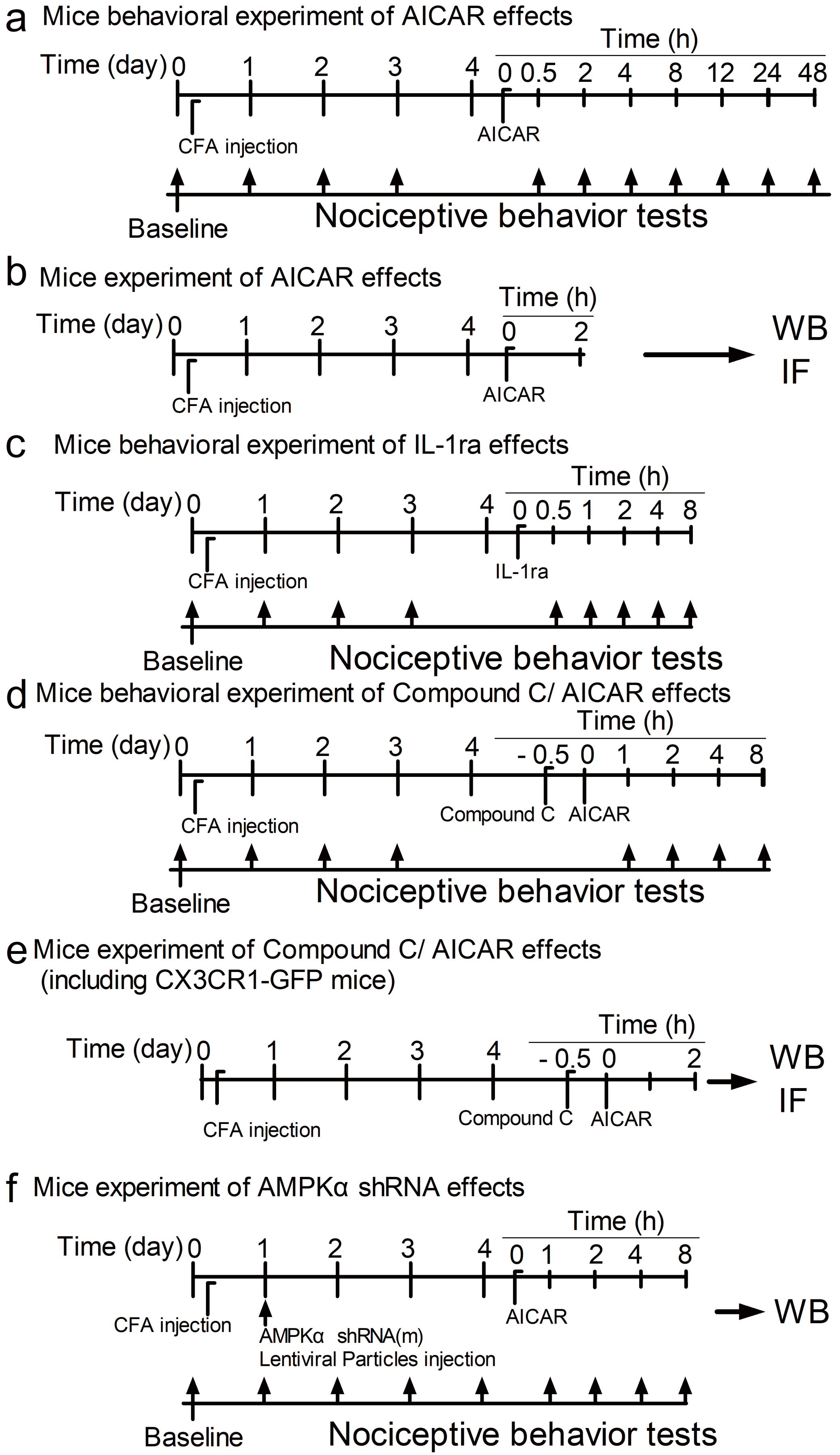

a Mice behavioral experiment of AICAR effects
Time (h)
Time (day) 0 1 2 3 4 0 0.5 2 4 8 12 24 48
CFA injection
AICAR
Baseline
Nociceptive behavior tests
b Mice experiment of AICAR effects
Time (h)
Time (day) 0 1 2 3 4 0 2
CFA injection
AICAR
WB
IF
c Mice behavioral experiment of IL-1ra effects
Time (h)
Time (day) 0 1 2 3 4 0 0.5 1 2 4 8
CFA injection
IL-1ra
Baseline
Nociceptive behavior tests
d Mice behavioral experiment of Compound C/ AICAR effects
Time (h)
Time (day) 0 1 2 3 4 - 0.5 0 1 2 4 8
CFA injection
Compound C
AICAR
Baseline
Nociceptive behavior tests
e Mice experiment of Compound C/ AICAR effects
(including CX3CR1-GFP mice)
Time (h)
Time (day) 0 1 2 3 4 - 0.5 0 2
CFA injection
Compound C
AICAR
WB
IF
f Mice experiment of AMPKα shRNA effects
Time (h)
Time (day) 0 1 2 3 4 0 1 2 4 8
CFA injection
AMPKα shRNA(m)
Lentiviral Particles injection
AICAR
WB
Baseline
Nociceptive behavior tests
